# 53BP1 expression is a modifier of the prognostic value of lymph node ratio and CA 19–9 in pancreatic adenocarcinoma

**DOI:** 10.1186/1471-2407-13-155

**Published:** 2013-03-26

**Authors:** Natalie L Ausborn, Tong Wang, Sabrina C Wentz, Mary Kay Washington, Nipun B Merchant, Zhiguo Zhao, Yu Shyr, Anuradha Bapsi Chakravarthy, Fen Xia

**Affiliations:** 1 Department of Radiation Oncology, Vanderbilt University School of Medicine, Nashville, TN, USA; 2 Department of Pathology, Vanderbilt University School of Medicine, Nashville, TN, USA; 3 Department of Surgery, Vanderbilt University School of Medicine, Nashville, TN, USA; 4 Department of Biostatistics, Vanderbilt University School of Medicine, Nashville, TN, USA; 5 Department of Radiation Oncology, The Ohio State University College of Medicine, Starling Loving, 300 W 10th Avenue, Columbus, OH, 43210, USA

**Keywords:** BRCA1 protein, 53BP1, Pancreatic cancer, DNA damage, Repair

## Abstract

**Background:**

53BP1 binds to the tumor suppressor p53 and has a key role in DNA damage response and repair. Low 53BP1 expression has been associated with decreased survival in breast cancer and has been shown to interact with several prognostic factors in non-small cell lung cancer. The role of 53BP1 in pancreatic ductal adenocarcinoma (PDAC) has yet to be determined. We aimed to investigate whether 53BP1 levels interact with established prognostic factors in PDAC.

**Methods:**

106 patients for whom there was tissue available at time of surgical resection for PDAC were included. A tissue microarray was constructed using surgical specimens, stained with antibodies to 53BP1, and scored for expression intensity. Univariate and multivariate statistical analyses were performed to investigate the association between 53BP1 and patient survival with known prognostic factors for survival.

**Results:**

The association of 53BP1 with several established prognostic factors was examined, including stage, tumor grade, surgical margin, peripancreatic extension, lymph node ratio (LNR), and CA 19–9. We found that 53BP1 modified the effects of known prognostic variables including LNR and CA 19–9 on survival outcomes. When 53BP1 intensity was low, increased LNR was associated with decreased OS (HR 4.84, 95% CI (2.26, 10.37), *p*<0.001) and high CA19-9 was associated with decreased OS (HR 1.72, 95% CI (1.18, 2.51), *p*=0.005). When 53BP1 intensity was high, LNR and CA19-9 were no longer associated with OS (*p*=0.958 and *p*=0.606, respectively).

**Conclusions:**

In this study, 53BP1, a key player in DNA damage response and repair, was found to modify the prognostic value of two established prognostic factors, LNR and CA 19–9, suggesting 53BP1 may alter tumor behavior and ultimately impact how we interpret the value of other prognostic factors.

## Background

Pancreatic ductal adenocarcinoma (PDAC) is the fourth leading cause of cancer death in the United States and continues to have a dismal prognosis, with a 5-year overall survival rate less than 4% [1]. The only potential curative option is surgical resection, for which less than 20% of patients are eligible. Even in this subset of patients, the 5-year overall survival remains only 18–24% [2-6]. Given the poor survival with surgery alone, attempts have been made to improve outcomes with adjuvant therapy. In multiple retrospective studies, chemoradiation therapy has been shown to confer a survival advantage compared with surgical resection alone [2,7,8]. Other studies suggest a benefit to adjuvant chemotherapy alone and not to adjuvant chemoradiation therapy [9-11]. The role of adjuvant therapy in the management of localized pancreatic cancer remains controversial as many of the randomized clinical trials were statistically underpowered and used outdated radiation fractionation schema and techniques. Therefore, tumor biomarkers that could be used to predict which subset of patients is likely to benefit from adjuvant therapy would be useful for clinicians to tailor therapy based on that individual patient’s tumor characteristics.

Many chemotherapeutic agents act by inducing DNA damage within rapidly dividing tumor cells. Ionizing radiation also causes DNA damage by inducing double-strand breaks (DSBs), which results in cell death. Molecular targets that regulate DNA damage and repair are hence likely to be good predictors of prognosis and response to treatment. The p53 binding protein 1 (53BP1) is a key mediator of DNA damage response, as it is a critical transducer of the DNA damage signal to p53 and other tumor suppressors [12]. DNA DSBs are typically repaired by two major pathways: template-based homologous recombination (HR) and nontemplate-based nonhomologous end joining (NHEJ) [13]. 53BP1 plays a key role in promoting the use of NHEJ to repair lethal DSBs [14]. Poly-(ADP-ribose) polymerase (PARP) is a key nuclear enzyme in DNA single-strand break repair whose inhibition induces synthetic lethality in BRCA1-mutated tumor cells which are HR deficient [15,16]. DNA double-strand breaks in BRCA1^Δ11/Δ11^ cells, which are deficient in HR, are aberrantly joined by NHEJ dependent on 53BP1 [17]. Interestingly, deletion of 53BP1 in BRCA1^Δ11/Δ11^ cells restored HR and alleviated hypersensitivity of *BRCA1*-mutated cells to PARP inhibition, rendering BRCA1^Δ11/Δ11^ cells insensitive to PARP inhibition [17]. In another study, Bouwman *et al*. found that 53BP1 deletion reversed cisplatin sensitivity induced by BRCA1 inactivation [18]. Thus, 53BP1 expression may be a good predictor of pancreatic tumor response to DNA damage-based therapy.

Additionally, low 53BP1 expression has been shown to be associated with poor prognosis. Bouman et al. found that loss of 53BP1 was associated with poor prognosis in patients with triple-negative breast cancer [18]. The role of 53BP1 in PDAC is yet to be determined. In this study, we investigate whether 53BP1 protein expression level is associated with pancreatic tumor behavior and how it interacts with other established prognostic factors in PDAC.

## Methods

### Patient selection and data collection

This study was approved by the Vanderbilt University Medical Center Institutional Review Board. 106 patients were identified who had undergone curative resections for pancreatic adenocarcinoma and for whom both clinical data and tumor tissue were available. Only patients with histologically confirmed ductal adenocarcinomas were included. All tumors were restaged by a single pathologist (SCW) according to AJCC 7^th^ edition criteria [19]. Data collected included patient demographics, operative details, treatment details and survival. Pathologic data obtained included tumor location, total number of nodes involved, total number of nodes resected, tumor size, differentiation, pancreatic extension, and margin status. A positive margin was defined as tumor within 1 mm of the inked resection margin on microscopic examination. The assessment of margins and slicing techniques were performed in a standardized fashion for all patients as previously described [20]. Tumor differentiation was recorded according to the guidelines outlined by the College of American Pathologists [21]. The lymph node ratio (LNR) was defined as the number of positive lymph nodes as a fraction of the total number of lymph nodes examined. CA 19–9 level was determined by a quantitative electrochemiluminescent immunoassay (Roche Modular E170) with a reference interval of 0–37 U/mL.

### Construction of tissue microarray

Tissue microarrays were constructed using 1 mm cores of both tumor and background normal/reactive pancreas from 106 curative resection specimens, including pancreaticoduodenectomy/gastrojejunostomy procedures (Whipple procedures) and total or distal pancreatectomies. The microarrays were composed of single or duplicate cores from tumor and background pancreas. The microarrays were cut at 5 μm-thickness and stained with hematoxylin and eosin.

### Immunohistochemistry study

5 μm-thick sections of formalin-fixed, paraffin-embedded tissue microarrays were de-paraffinized and rehydrated. Samples were pretreated to promote antigen retrieval with Target Retrieval Solution (DAKO, Carpinteria, CA, USA). Sections were blocked with 3% hydrogen peroxide, followed by blocking in 2% goat serum/0.1% Triton-X 100/PBS (1 hour). Slides were then incubated with primary antibody 53BP1 (1:200 dilution in blocking buffer, NOVUS, Cat. No. NB100-304) overnight at 4°C. Slides were washed in phosphate-buffered saline. DAB (3,3-diaminobenzidine tetrahydrochloride dehydrate, Invitrogen, Cat.  No. 00–2020) was applied. 53BP1 expression was analyzed by microscopy (Carl Zeiss, Thornwood, NY).

**Figure 1:**
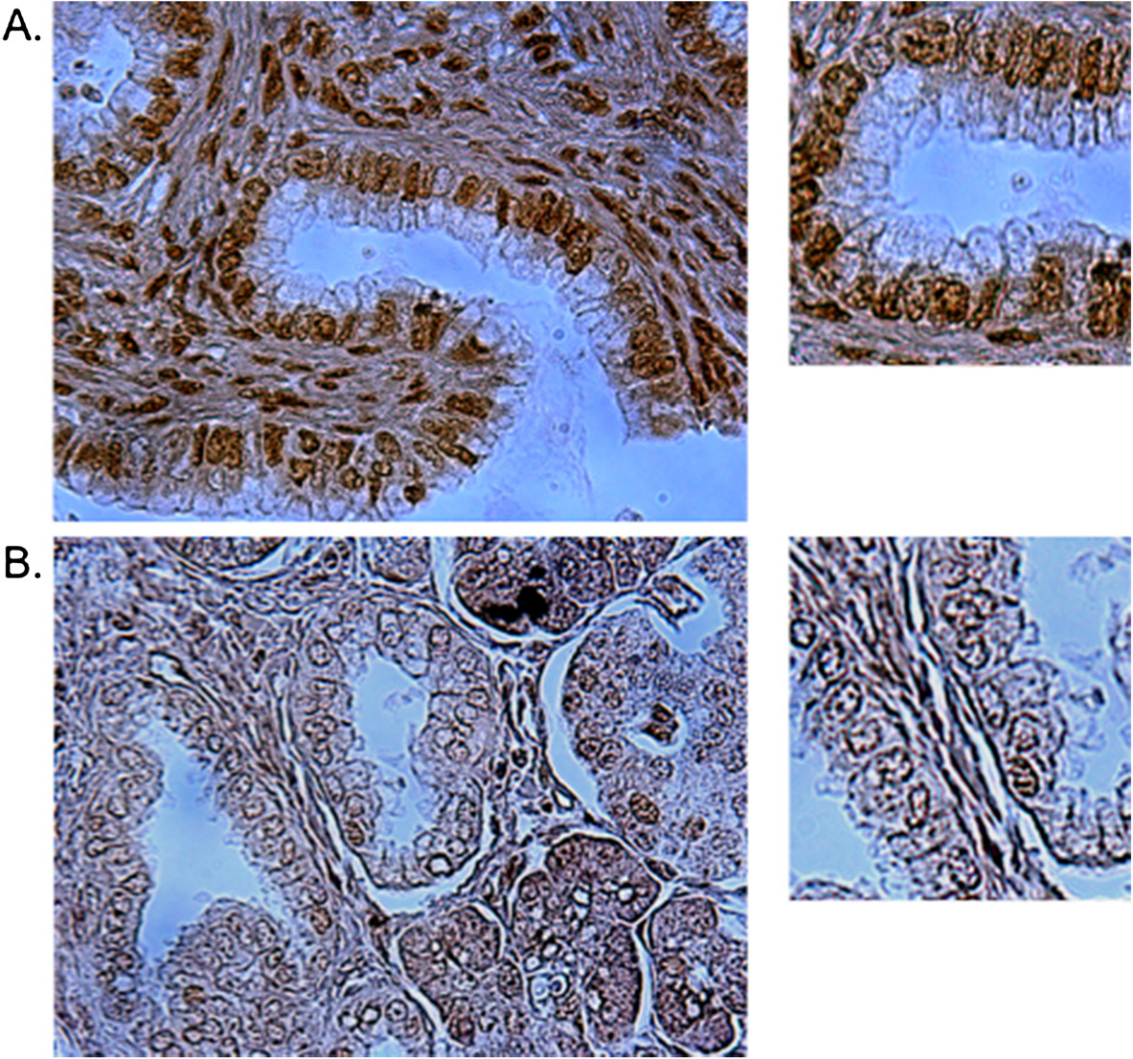
**Representative immunohistochemistry staining for 53BP1 expression in pancreatic adenocarcinoma.** (**A**) is high intensity of 53BP1 expression and (**B**) is low intensity of 53BP1 expression.

Positive and negative controls were established with the use of human breast cancer cells and a human breast tumor specimen. The MCF-7 cell line, which is derived from human breast cancer and expresses 53BP1 as confirmed by us and other research laboratories [22], was first used to perform 53BP1 antibody immunostaining. The MCF7 cells were not paraffin imbedded and were instead cultured as a monolayer in a tissue culture chamber prior to immunostaining. For a positive control, cells were stained with antibody to 53BP1 as described above. For a negative control, the primary antibody was not used and instead was replaced by non-immune rabbit IgG at the same concentration of the primary antibody. We obtained positive staining in the positive control and negative staining in the negative control. Next, 53BP1 immunostaining was performed in a human cancer tumor specimen. A human breast tumor specimen was used because breast tumors have been reported to have positive 53BP1 expression [23,24]. 5 μm-thick consecutive sections of formalin-fixed, paraffin-embedded breast cancer tissue were de-paraffinized and rehydrated as described above. Tissue was stained with antibody to 53BP1. For a negative control, a consecutive section from the same specimen was not stained with the primary 53BP1 antibody and instead was replaced by non-immune rabbit IgG at the same concentration of the primary antibody. The immune-staining specificity of the 53BP1 antibody was confirmed by no staining in the negative control which omitted the primary 53BP1 antibody and positive staining in the specimen to which the primary antibody was applied. For each pancreatic cancer tissue microarray, a total of 300–450 cells were counted. The intensity of staining was scored as 1+ (weakly staining/least intense), 2+ (moderately staining), or 3+ (strongly staining/most intense) in tumor cells. Due to limited sample size, 1+ and 2+ staining intensity were grouped into “low” intensity, and 3+ staining intensity was referred to as “high” intensity. Stromal cells were not included in the scoring. Assessment of 53BP1 staining was performed by a single person blinded to patient outcomes. Representative immunohistochemical staining is shown in Figure 1.

### Statistical analysis

The primary endpoint was defined as the time from surgery to the date of all-cause death (overall survival, OS) or last follow-up. The secondary endpoint was defined as the time from surgery to the date of disease recurrence (recurrence-free survival, RFS) or last follow-up. Patients’ demographic and clinical variables were summarized using the median with the 25th and 75th percentiles (interquartile range, IQR) for continuous variables. For categorical variables, frequency and percentages were shown. The Wilcoxon rank sum test was used for continuous variables, and Pearson’s *χ*2 or Fisher’s exact test was used to compare categorical variables between 53BP1 intensity groups (low or high). The Kaplan-Meier method, Log-rank test, likelihood ratio test, and Cox proportional hazard (Cox PH) models were used in univariate and multivariate analysis when appropriate to investigate the associations between the endpoints and the risk factors. Predetermined interactions of 53BP1 levels by LNR and 53BP1 levels by CA 19–9 levels were included in all multivariable models. All statistical inferences were assessed at a two-sided 5% significant level and all summary statistics, graphics, and survival models were generated using R version 2.13 statistical software [25].

## Results

### Patient clinical and pathologic characteristics

106 patients were identified who had undergone curative resections for PDAC for whom tissue samples were also available for study. Table 1 summarizes the demographic and treatment details as well as clinicopathologic findings. Of the 106 patients undergoing surgical resection, 71.0% had microscopically negative surgical margins. The median OS for all patients was 15.5 (IQR: 8.2–35.4) months.

**Table 1:** Patient clinical and pathologic characteristics

	***N***	**No. (%)**
**Age,** years	106	68 (58–73) ^a^
**Gender**	106	
Female		47 (44)
Male		59 (56)
**Race**	105	
African American		6 (5.7)
Caucasian		99 (94.3)
**Tumor grade**	106	
1		15 (14)
2		62 (58)
3		29 (27)
**Tumor stage**	106	
I-IIA		31 (29)
IIB-IV		75 (71)
**Tumor size**	106	3.0 (2.1–3.5) ^a^
**Lymph node status (N stage)**	104	
N0		35 (34)
N1		69 (66)
**Number of resected nodes**	104	11.0 (8.0–18.2) ^a^
**Number of positive nodes**	104	1.0 (0.0–4.0) ^a^
**Lymph node ratio**	104	0.095 (0.000–0.257) ^a^
**Operation type**	106	
Whipple		93 (87.7)
Distal pancreatectomy		9 (8.5)
Total pancreatectomy		3 (2.8)
En bloc resection		1 (1)
**Surgical margin status**	106	
Negative		75 (71)
Positive		31 (29)
**Adjuvant chemotherapy**	102	
No		27 (26)
Yes		75 (74)
**Adjuvant radiation therapy**	102	
No		50 (49)
Yes		52 (51)
**CA 19-9**	106	157 (50–520) ^a^
**53BP1 expression intensity**	106	
Low intensity		62 (58)
High intensity		44 (42)
**Overall survival status**	106	
Alive		23 (22)
Deceased		83 (78)
**Survival time,** months	106	15.5 (8.2–35.4) ^a^

^a^: median (IQR).

### Univariate analysis

53BP1 staining intensity was not found to be associated with OS or RFS (*p*>0.05). 53BP1 intensity was not found to be associated with tumor stage, tumor grade, adjuvant chemotherapy use, CA 19–9 level, or LNR (*p*>0.05). Findings are summarized in Table 2.

**Table 2:** Univariate analysis of 53BP1 expression intensity

	**Low intensity ( *N=62 *), No. (%)**	**High intensity ( *N=44 *), No. (%)**	***p*-value**
**Tumor grade**			0.85 ^a^
1	8 (13%)	7 (16%)	
2	36 (58%)	26 (59%)	
3	18 (29%)	11 (25%)	
**Tumor stage**			0.98 ^a^
I-IIA	18 (29%)	13 (30%)	
IIB-IV	44 (71%)	31 (70%)	
**Adjuvant chemotherapy**			1 ^a^
No	16 (27%)	11 (26%)	
Yes	44 (73%)	31 (74%)	
**CA 19-9**	210 (66–660) ^1^	112 (32–337)^1^	0.12 ^b^
**Lymph node ratio**	0.10 (0.00–0.25) ^1^	0.09 (0.00–0.27) ^1^	0.92 ^b^

^a^: Fisher’s Exact Test.

^b^: Wilcoxon Rank Sum Test.

^1^: Median (IQR).

### Multivariable analysis

Recent molecular correlative studies in glioblastoma have shown that certain molecules can modify the value of other prognostic biomarkers [26]. Although 53BP1 intensity was not associated with any of the known prognostic factors in univariate analysis, we postulated that 53BP1 expression levels could interact with and thereby modify established prognostic markers in PDAC. Lymph node involvement in PDAC predicts for worse survival. Carbohydrate 19–9 antigen (CA 19–9) is a sensitive and specific biomarker for pancreatic cancer. The total CA 19–9 values as well as the rates of decline have been shown to predict survival in patients with advanced disease [27-31].

**Figure 2:**
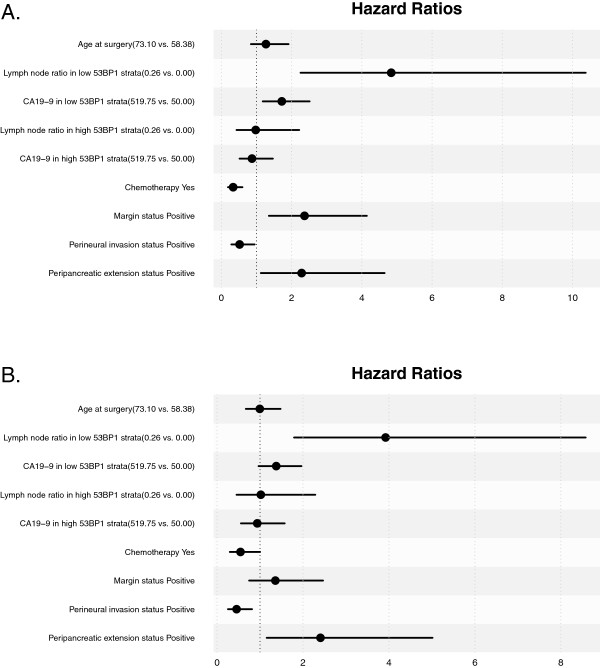
Hazard ratios for clinical and pathologic characteristics for (A) OS and (B) RFS.

In multivariable analysis, we observed that 53BP1 intensity modifies the prognostic ability of both LNR and CA19-9. When 53BP1 intensity is low, increased LNR was associated with decreased OS (HR 4.84, 95% CI (2.26, 10.37), *p*<0.001) and high CA19-9 was associated with decreased OS (1.72, (1.18, 2.51), *p*=0.005). When 53BP1 intensity is high, LNR and CA19-9 were not associated with OS (*p*=0.958 and *p*=0.606, respectively). When 53BP1 intensity was low, increased LNR was also associated with decreased RFS (3.92, (1.79, 8.58), *p*<0.001) and high CA 19–9 had a trend towards decreased RFS that did not reach statistical significance (1.38, (0.97, 1.96), *p*=0.077). Results are summarized in Table 3. Hazards ratios are shown in Figure 2.

**Table 3:** Multivariable Cox PH analyses for OS and RFS

	**Overall survival**			**Recurrence free survival**		
**Variables**	**HR**	**95% CI**	***p *value**	**HR**	**95% CI**	***p *value**
Age at surgery ^1, a^	1.27	0.84–1.91	0.255	1.00	0.67–1.48	0.982
Lymph node ratio			0.007 ^b,*^	1.02		0.020 ^b,*^
Within High 53BP1 level strata ^2, a^	0.98	0.43–2.22	0.958	3.92	0.45–2.29	0.963
Within Low 53BP1 level strata ^2, a^	4.84	2.26–10.37	<0.001^*^		1.79–8.58	<0.001^*^
CA19-9			0.040 ^b,*^			0.226 ^b^
Within High 53BP1 level strata ^3, a^	0.87	0.52–1.47	0.606	0.94	0.56–1.57	0.803
Within Low 53BP1 level strata ^3, a^	1.72	1.18–2.51	0.005^*^	1.38	0.97–1.96	0.077
Adjuvant chemotherapy: Yes	0.34	0.19–0.60	<0.001^*^	0.55	0.30–1.00	0.051
Margin Positive	2.37	1.35–4.14	0.003^*^	1.36	0.75–2.47	0.316
Peripancreatic extension status Positive	2.29	1.12–4.65	0.022^*^	2.41	1.16–5.01	0.019^*^
Perineural invasion status Positive	0.52	0.29–0.95	0.033^*^	0.46	0.25–0.81	0.008^*^

^a^: upper quartile vs. lower quartile.

^b^: P value for interaction terms with 53BP1 Level.

^1^: 73.10 vs. 58.38.

^2^: 25.69% vs. 0%.

^3^: 519.75 vs. 50.

^*^: statistically significant.

## Discussion

In this study, 53BP1 was found to modify the effect of two established pancreatic cancer clinicopathological prognostic factors, LNR and CA 19–9 level, on patient survival.

The number of nodes involved is a function not only of the rates of true node positivity but also of the aggressiveness of the surgeon in obtaining these nodes and the pathologist in finding these nodes at the time of resection. Adequate staging of node negative pancreatic cancer requires the evaluation of at least 12 nodes. Unfortunately, this is not always feasible. The ratio of number of positive nodes to the number of nodes examined or the LNR help to equalize these variations in both surgical technique as well as pathological nodal evaluation. LNR has been shown to be of prognostic value in a variety of gastrointestinal tumors including cancers of the stomach, esophagus, colon, rectum, and biliary tract [32-36] and has been suggested as an important prognostic factor in pancreatic cancer as well [37-40]. However, the current determination of N stage in pancreatic cancer is delineated as either positive or negative, rather than the absolute number of nodes or LNR. In our study, we found that when 53BP1 intensity was low, increased LNR was associated with decreased OS (HR 4.84, *p*<0.001) and RFS (HR 3.92, *p*<0.001). However, the association of LNR with survival was lost when 53BP1 intensity was high, suggesting that 53BP1 may modulate the tumor cellular environment where low 53BP1 expression level causes worse prognosis for high LNR.

CA 19–9, a sialylated Lewis (Le^a^) antigen of the MUC1 protein, is another prognostic marker in pancreatic cancer, and serial measurements of CA 19–9 have been shown to be useful to monitor treatment response [41,42]. There are several studies that have evaluated CA 19–9 as a pretreatment prognostic marker [27-31], and although there is no established threshold value for prognostic evaluation, 370 U/ml has been found to divide patients into two groups with a significant difference in survival [43]. In our study, we found that when 53BP1 intensity was low, high CA19-9 was associated with decreased OS (HR 1.72, p=0.005). When 53BP1 intensity was high, CA 19–9 was no longer associated with overall survival.

Taken together, our results suggest 53BP1 expression levels may precondition the tumor cell biological behavior. 53BP1, as a DNA damage response protein, is thought to promote NHEJ and suppress HR [14]. Increased 53BP1 levels therefore likely allows efficient cellular repair of endogenous DNA damage in response to metabolic stress or chemotherapy and radiation therapy; however, when 53BP1 levels are decreased, there is decreased NHEJ efficiency to repair DNA damage. Our data suggest that low 53BP1 expression may predispose pancreatic tumor cells to become more vulnerable to changes of intrinsic metabolic stress, tumor microenvironment, and genotoxic stress from DNA damage based therapy. In turn this could modify how other prognostic factors such as LNR and CA19-9 predict overall survival.

In a cohort of breast cancer patients treated with breast-conserving surgery and radiotherapy, low 53BP1 was associated with worse clinical outcomes including recurrence-free survival, distant metastasis-free survival, and overall survival [24]. Bouman *et al.* found that 53BP1 loss was associated with the poor prognosis triple-negative breast cancers [18]. 90.5% of breast tumors that were deficient in 53BP1 were triple-negative. Of the triple-negative tumors assayed, 43% were 53BP1 negative and in non-triple-negative tumors, only 2% were 53BP1 negative (*p*<0.0001). Together, this data suggests 53BP1 loss is more frequent in more aggressive breast cancers. While in our study low 53BP1 was not directly associated with overall survival, low 53BP1 expression modified the prognostic value of CA 19–9 and LNR so that high CA 19–9 and high LNR were associated with worse OS. With high 53BP1, LNR and CA 19–9 were no longer associated with overall survival. One study has shown an association between 53BP1 and established lung cancer prognostic factors, such as smoking status, lymphovascular invasion, and tumor stage [44].

Due to study size limitation, our study was unable to test whether 53BP1 could modify the effects of other clinicopathological factors such as adjuvant chemotherapy, margin status, peripancreatic extension status, and perineural invasion status. For example, based on the biologic function of 53BP1, 53BP1 may modify the prognostic value of adjuvant chemotherapy such as the use of PARP inhibitors due to the ability of 53BP1 to alter homologous recombination (HR) and nonhomologous end joining (NHEJ). Loss of this protein may result in the inability of cells to repair damaged DNA and modify sensitivity to chemotherapeutic agents. Also, an important question not addressed in our study that should be addressed in a larger study is the relationship between any of the clinicopathologic factors and local recurrence or distant metastasis.

There are several limitations to our study. For instance, our study group is heterogenous in that patients were included regardless of type of surgical procedure, and the number of lymph nodes retrieved may vary considerably among those procedures. In order to increase our sample size all patients were included. Additionally, in our study perineural invasion was found to have a positive impact on survival, which is inconsistent with the literature. Our finding may be the result of small numbers and the retrospective nature of tissue collection. Our study found that CA 19–9, positive margin, and adjuvant chemotherapy were associated with OS but not RFS. The lack of association with RFS may be a function of the difficulty of accurately coding of recurrence in a retrospective study that spanned such a long time period. Therefore, our hypothesis should be tested with prospectively collected tissue in a large cooperative group setting.

Future studies are warranted to further characterize the role of 53BP1 in PDAC as well as to study the mechanisms by which 53BP1 intensity affects tumor cell behavior. Our results point to the complexity of the translation of cancer cell biology to clinical tumor behavior. A hallmark of cancer cells is the possession of multiple gene mutations and aberrations in cell signaling pathways. The ability to identify a single biomarker to predict tumor response has been disappointing. There will always be an interaction with additional biomarkers or clinicopathological factors. Therefore, it is necessary to interpret the predictive value of a particular biomarker in light of the status of other biomarkers in that individual tumor. Stratification of tumors based on the summation of several biomarkers and clinicopathological factors will allow for better predictive value in the clinic. As the role of 53BP1 in tumors has been shown in several studies to modify the sensitivity of BRCA-mutated cells to chemotherapeutic agents (PARP inhibitors, cisplatin) [17,18], future studies examining the role of 53BP1 in BRCA-mutated pancreatic cancer would be of clinical value.

## Conclusion

In summary, our results demonstrate 53BP1 modifies the effect of two established pancreatic prognostic factors, LNR and CA 19–9, suggesting 53BP1 may alter tumor behavior and ultimately impact how we interpret the clinical value of other prognostic factors.

## Abbreviations

PDAC: Pancreatic ductal adenocarcinoma; DSBs: Double-strand breaks; 53BP1: P53 binding protein 1; HR: Homologous recombination; NHEJ: Nonhomologous end joining; PARP: Poly-(ADP-ribose) polymerase; LNR: Lymph node ratio; OS: Overall survival; RFS: Recurrence-free survival; IQR: Interquartile range; CA 19–9: Carbohydrate 19–9 antigen.

## Competing interests

Nipun Merchant has received honoraria from Covidien and Medtronics 2012 and Advisory Board for Biocompatibles, Inc., 2012. Fen Xia has received honoria from Abbott, 2011. All remaining authors have declared no conflicts of interest.

## Authors’ contributions

NLA carried out data collection, assisted in data interpretation, and drafted the manuscript. TW carried out immunohistochemistry and assisted in data collection. SCW determined tumor staging and assisted in data collection. MKW participated in the data collection and coordination of the study. NBM assisted in coordination of the study and revision of the manuscript. ZZ and YS performed the statistical analysis and aided in data interpretation. ABC participated in the design of the study, data interpretation, and revision of the manuscript. FX conceived of the study, participated in its design and coordination, and helped to draft and revise the manuscript. All authors read and approved the final manuscript.

## Pre-publication history

The pre-publication history for this paper can be accessed here:

http://www.biomedcentral.com/1471-2407/13/155/prepub
